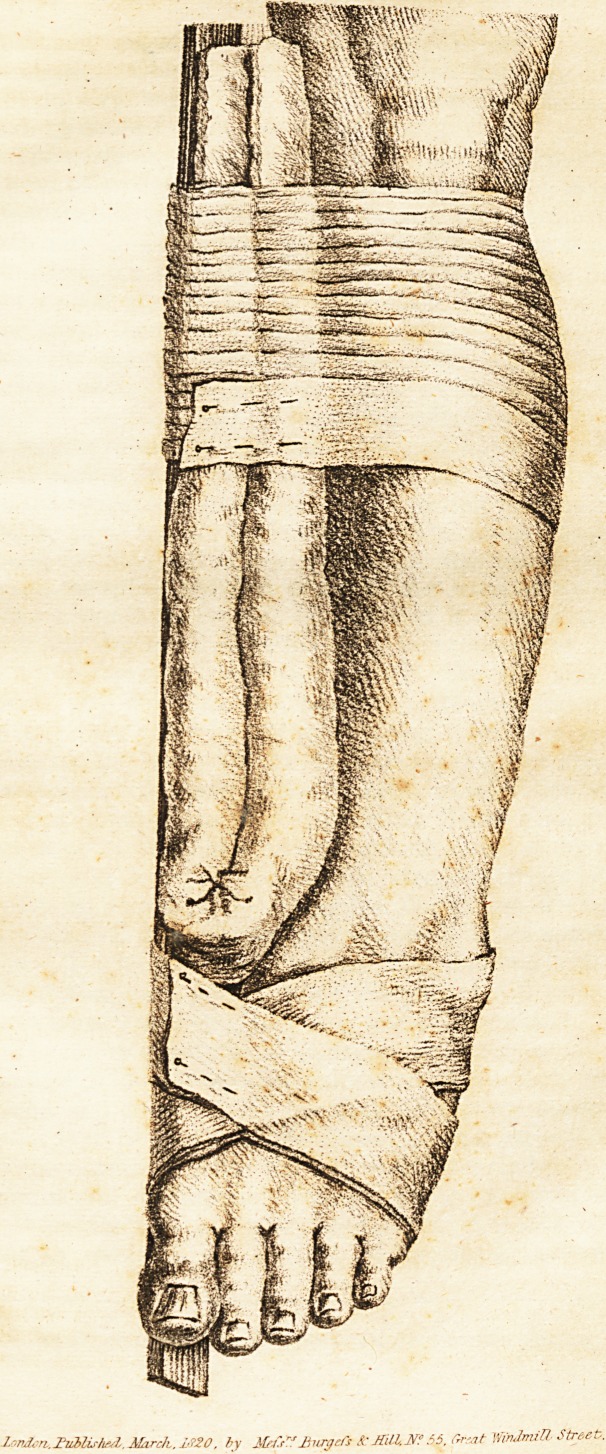# Medico-Chirurgical Annals of the Parisian Hospitals

**Published:** 1820-06-01

**Authors:** 


*Annuaire Medico-Chirurgicale des Hôpitaux et Hospices civils de Paris; ou Recueil de Memoires et Observations, par les Medecins et Chirurgiens de ces Etablissemens.* Quarto with plates, pp. 636. Paris, 1820, Vol. I. viz.

*Medico-Chirurgical Annals of the Parisian Hospitals; or Repository of Memoirs and Observations, furnished by the Physicians and Surgeons of those Establishments.* Vol. I.

This work is similar, in plan and design, to the Dublin Hospital Reports, the Transactions of the Irish Association, and, in some measure, to the Medico-Chirurgical Transactions of London. It is superintended by a deliberative council, and appears to be restricted to the medical officers designated in the title page. The medical profession are too well convinced of the great value of such works, to require any preliminary observations from us; and therefore we shall proceed at once to the execution of our analytical duties.

Art. 1. *On Fracture of the lower Extremity of the Fibula, and the Luxations and Accidents accompanying it.* By M. Dupuytren, Surgeon in Chief to the Hôtel Dieu, &c.

This is a most extended and important article, embracing the history of fractures and dislocations of the ancle-joint; together with the anatomy and physiology of that part. We shall endeavour to give a pretty full analysis of this paper.

Among the accidents resulting from *perpendicular falls* on the feet, M. Dupuytren presents us with the following interesting case.

"A man, 50 years of age, jumped from the top of a coach in motion, and pitched on the left heel with all his weight. He immediately experienced intense pain in the ancle joint; and Dr. Nauche being summoned, found a hard, prominent, unequal tumour under the skin, and anterior to the lower extremities of the tibia and fibula. It was irreducible, and accompanied with much surrounding extravasation. Dupuytren being called in, recognized a dislocation of the astragalus, which had been driven from its place by the pressure of the tibia, in falling. What was to be done? No force could reduce the bone to its place. They determined to cut it out. An incision, parallel to the axis of the foot, was made over the tumour. On laying the bone bare, and endeavouring to extract it, they were foiled, and found to their surprise, that the astragalus was completely inverted, its under surface being uppermost, in consequence of which its salient part was hooked in under the tibia, and offered the resistance above mentioned. By means of a strong iron wire passed round the neck of the astragalus, that bone was happily extracted. Lint and emollient poultices were applied to the wounded limb, which was laid in an easy position; repeated bleedings were practised; rigid abstinence enjoined. Inflammation and high fever followed; suppuration took place; but the man ultimately recovered perfectly, with, of course, an anchylosed joint." 28.

M. Dupuytren made a great number of experiments on the dead subject, in order to ascertain the nature of dislocations of the ancle-joint, some of the results of which we shall present to the English reader.

1mo. A gentle twist of the foot inwards or outwards, produces only a slight distention of the ligaments, as in a common strain. A greater effort of this kind produces a separation of the ligaments, *at their attachment to the malleoli,* either by tearing asunder their compact tissue, or by stripping off the periosteum which covers the malleoli, leaving the ligaments themselves unbroken; a mode which most frequently takes place in the living subject.

Let the twist, or unnatural flexion of the joint, be still more sudden and violent, then not a rupture of ligaments, as was long supposed, but a fracture of the malleoli themselves takes place. When the foot is thus violently bent *outwards*, a rupture of the internal lateral ligaments, or fracture of the *internal* malleolus, always precedes the fracture of the fibula. When the foot is contorted *inwards*, the fibula is almost always fractured, while the internal malleolus, and corresponding lateral ligaments remain whole. In both cases, the malleoli are broken by the strain of the ligaments coming on the extreme points of these bones, and thus snapping them off. The fibula, however, may be fractured by the whole weight of the body coming upon it, after the internal ligaments or malleolus have given way in violent contortions of the foot *outward*. Our author here remarks on the importance of attending to *strains* of the ancle-joint, by rest bandages, &c. Since nothing renders us so liable to fractures of the malleoli, as the relaxed state of the ligaments consequent on a neglected strain.

In accidents of the ancle-joint, a fracture of the fibular malleolus is by no means always so apparent as to leave no doubt upon the subject, especially if some time have elapsed between the infliction of the injury and seeing the patient. M. Dupuytren has therefore enlarged on the presumptive and characteristic signs of the fracture in question.

Among the *presumptive* signs are, a sensation, by the patient himself, of something having snapped; a fixed pain at the lower extremity of the fibula; a difficulty, or even impossibility of walking; a greater or less degree of swelling round the joint, especially about the outer malleolus. The *characteristic* symptoms are, inequalities; an unnatural degree of mobility at some point of the fibular extremity; more or less crepitation; an unusual degree of *lateral* motion in the foot, which appears thrown a little outwards, sometimes backwards; an angular depression, more or less marked, above the outer ancle; prominency of the inner ancle. Finally, a disappearance of all these symptoms, when the foot is properly extended and placed in its natural position, and a return of them all again, so soon as the foot and muscles of the limb are left to themselves, without splints or bandage.

M. Dupuytren introduces the following case to shew that the above phenomena, isolated or combined, are not always present, and consequently will not always indicate to us the real state of things, in accidents of the ancle-joint.

"*Case.* A man, walking along a narrow road, stumbled, and fell with his right leg bent under him. He felt an acute pain in the ancle at the moment, and he was unable to put it to the ground. He was therefore carried home, and M. Dupuytren was called to him eight or ten hours after the accident. The foot and leg appeared perfectly natural, nor was there any unnatural degree of mobility in the joint in any direction. The malleoli exhibited no symptom of any solution of continuity; and when the limb was in a state of semi-flexion, no pain was experienced. Nevertheless, the patient could not bear in the slightest degree upon the foot, without feeling exquisite pain above the outer ancle. Pressure with the finger also upon this point caused a similar suffering. There was now some ecchymosis along the lower part of the fibula; but no crepitation on moving the foot, nor distortion of it from its natural position. M. Dupuytren, nevertheless, pronounced the existence of a fracture of the fibula, at the place where the pain was felt; but as there was no displacement, it only required rest, discutients, and demi-flexion of the limb. Every thing was going on well, under this plan; when the patient was advised (by some other person) to get out of bed, and take a little gentle exercise, to improve his health and prevent stiffness of the joint! He did so; but scarcely had he put his foot to the ground, when something appeared to snap, accompanied by intense pain. He fell to the ground, and was unable to get up! M. Dupuytren being summoned, found the foot now turned outwards, with the mobility and crepitation before alluded to. The apparatus (which will be fully described towards the close of this article) was applied; rest, and the proper means enjoined; and a cure was effected in six weeks." 75.

We shall introduce the following case, not only as reuniting the more prominent symptoms attending this serious accident, but as illustrating an important point of practice, which will be particularly noticed in the progress of this analysis.

"*Case.* Jean T. 26 years of age, slipped, while walking on a wet pavement, and fell on her right side, with the limb bent under her. She experienced acute pain in the ancle, and tried to get up, but could not bear the least weight on the foot. She was carried the next day to the Hotel Dieu, and presented the following symptoms: — Foot turned outwards; rotation of the foot on its axis; considerable prominency of the tibia and malleolus internus, with much tension of the skin covering them, with phlyctenæ, filled with reddish serosity. On the opposite side there was a considerable depression, with transverse fold of the skin, about two inches above the external ancle. On the least effort at reduction all these symptoms disappeared; but were quickly reproduced when the limb was left to itself. To these characters of outward luxation were added, an acute pain at the lower part of the fibula; inequalities of surface; preternatural mobility; crepitation; such a facility of bending the foot from side to side, as might lead one to suppose both malleoli broken and their ligaments destroyed — incontestible signs of fractured fibula. There was considerable ecchymosis on both sides of the ancle, with tumefaction; great tension round the whole joint, accompanied by pain, which last was always moderated by bringing the foot into its natural position. The surgeon on duty only applied a cataplasm. The following day (two from the injury) M. Dupuytren found the tumefaction amounting to an œdematous swelling; and judging that all the bad symptoms were kept up by the dislocated state of the parts, determined on reducing the dislocation *without waiting for a subsidence of the tension and inflammation.* The limb therefore being bent, and the upper part of the leg fixed by an assistant; another assistant grasped the heel with one hand, while the other was applied over the instep. A slight and gentle extension of the foot was made, turning it a little inwards at the same time. It was easily brought to its natural position, the prominency and tension of the inner ancle disappeared, and the pain was much mitigated. The apparatus, hereafter to be described, was applied, the limb, half-bent, placed on its outer side, and antiphlogistic measures prescribed. There was considerable fever, much local inflammation, and, at one time, suspicion of abscess about the joint; but all did well, and the patient was able to walk in sixty days." 90, 1, 2.

M. Dupuytren here makes an important practical distinction between fractures which occur *within* three inches of the malleolus externus, and those which occur at *more* than that distance from the extremity of the fibula. In the *latter* case there is no danger of dislocation of the ancle, as the integrity of the tibio-peroneal ligaments is preserved. But where the fracture is within the above-mentioned distance of the maleolus internus, if dislocation do not take place at the time of the accident, it is liable to occur on the patient's making the least attempt to walk. This important fact ought to be graven on the memory of every surgeon. Dupuytren observes, that although the fibula is occasionally fractured at every point between its malleolar extremity and middle, yet the solution of continuity generally takes place about two and a half inches from the inferior extremity of the bone—a point where the fibula is weakest, and where there is a natural bend inwards from the weight of the body and habitual action of the muscles.

The fracture in question may be complicated with rupture of the lateral ligaments, and fracture of the internal malleolus. The following case is illustrative of this complication.

" *Case.* Louise Grandjean, 45 years of age, fell backwards, and towards the right side, on the 7th of September, 1809, having the left foot engaged between two stones. She experienced, at the moment, an acute pain at the inferior and outer part of the left leg, and found herself incapable of putting the foot to the ground. She was therefore carried home, and two days afterwards, sent to the Hotel Dieu. Notwithstanding the swelling of the ancle, a fracture of the lower extremity of the fibula was recognized by the following signs: viz. pain; depression; mobility; crepitation at the inferior and exterior part of the limb; declination of the foot outwards. To these were added, considerable ecchymosis and acute pain around the *inner* ancle; a prominency of the inferior extremity of the tibia, which appeared unequal, and shorter than natural, with great tension of the integuments. Below this prominency was felt a deep groove, and still lower, a hard moveable substance, which was in fact, the malleolus internus broken off from the tibia. Dupuytren's apparatus was applied for thirty-five days; at the end of which time, the fracture of the fibula was consolidated, as well as that of the malleolus internus. In three weeks more the patient walked, and the movements of the foot were free as before the accident." 107.

M. Dupuytren believes that fracture, with luxation of the foot *backwards*, that is, where the tibia is thrown forward towards the instep, is always produced by the action of the gastrocnemii muscles, and not by the same causes which produce the fracture of the fibula. This kind of luxation is always incomplete, while the malleolus internus remains unbroken; in which case, the foot is turned outwards, and at the same time backwards. But where the internal malleolus is fractured, which is often the case, the dislocation may then be to a great extent. The heel becomes lengthened, and a bony tumour projects under the tendons and ligaments in front. But, whereas, in simple luxations of the foot, the external malleolus follows the movements of the tibia and fibula, forming a prominency similar to that of the inner malleolus, it is, in the case now under consideration, dragged backwards with the foot, to which it adheres by the lateral ligaments.

It is in these cases that the great advantages of *demi-flexion* of the limb become evident. The *extended position* alone, reproduces the accident, after reduction, and is therefore injurious.

" *Case in Illustration of this Kind of Dislocation.* Pierre Fromont, 33 years of age, while carrying a heavy burthen, stumbled and fell in such a manner that his left foot was violently bent successively inwards and backwards. On his entrance into the Hotel Dieu, the 24th of June, 1817, the anterior part of the foot appeared so shortened, and the heel so elongated, that the leg seemed placed midway between the os calcis and toes. The fore part of the foot was depressed, and the heel drawn a little up. At the part corresponding with the instep, or anterior angle formed by the leg and foot, was a hard projection, with great tension of the extensor tendons. Behind the joint was a considerable hollow traversed by the tendo Achillis. The malleolus externus, dragged backwards, manifested a mobility and crepitation that denoted fracture of the fibula. The malleolus internus was very prominent, and contrasted with the outer ancle, in being so much further forwards. The joint was incapable of flexion or extension; but in causing the least lateral movement, the foot inclined outwards.

"It was evident that in this case, a fracture of the fibula had first taken place; then a luxation of the foot backwards; and that, last of all, the malleolus externus, adherent to the os calcis, had followed the movement of this bone backwards; while the tibia, remaining in its position, *appeared* to project inward and forward.

"The pain, tumefaction, tension, heat, and redness, were considerable, and every moment increasing. M. Dupuytren, persuaded that the surest method of checking these symptoms was by replacing the dislocated parts, proceeded at once to the reduction. The limb was bent—the superior part held by an assistant—extension and counter-extension made by M. Dupuytren himself, who soon succeeded in bringing all into their proper places. The apparatus was applied, and antiphlogistic measures pursued. In thirty-six days the patient was discharged cured." 113.

A species of fracture is next described by M. Dupuytren, in which the astragalus is carried under the malleolus externus, while the outer edge of the foot is turned downwards, the sole of the foot inwards, and the inner edge of the foot upwards. In this case, the inner malleolus is concealed in a hollow angle between the foot and the leg, while the malleolus externus with the astragalus, forms a salient angle on the opposite side; in short, the whole appears like a congenital club-foot. This species of dislocation is not of very frequent occurrence. The following case, in illustration, is very interesting.

"Mademoiselle M——, about 50 years of age, fell from a balcony on the first floor, into the street, and pitched upon the internal edge of the right foot and inner ancle. When lifted up, she felt an acute pain in the articulation, but without any appearance of dislocation. Being carried into her apartment she attempted to bear upon the injured limb, but instantly fell to the ground, from the violence of the pain. M. Dupuytren was called in; and *now* the foot was much turned inwards, the malleolus externus forming a salient angle. The internal surface of the foot and ancle formed a hollow, while the opposite side was bulged out in the form of a semi-circle. In the concavity, that is, on the inside of the limb, I felt under the skin, and notwithstanding the ecchymosis and swelling, a prominent knob, making part with the malleolus internus—'un bec saillant qui faisait corps avec la malléole interne.' A crepitation was perceptible about the outer ancle, and somewhat anterior to this, a projection formed by the astragalus. There was, therefore, a fracture of the tibia as well as of the fibula; the former being oblique, extending from within outwards, and from above downwards.

"The reduction was easy; but a retention of the parts, in situ, was more difficult; for, as soon as the limb was left to the action of its own muscles, the foot was drawn inwards, and the astragalus projected exteriorly. Semi-flexion of the limb diminished this tendency to displacement, but did not entirely overcome it. The apparatus was therefore in this case applied to the *outside* of the leg, and all the indications were immediately fulfilled. She recovered." 118.

Another complication, still more unfrequent than the above, (M. Dupuytren having only met with one case out of two hundred admitted into the Hotel Dieu, or seen in private practice, during fifteen years), is where the foot is dislocated *outwards* and at the same time *upwards*. It cannot take place without fracture of the fibula, and complete laceration of the tibio-peroneal ligaments. The following case will illustrate this dislocation.

"Charles Nicholas, 54 years of age, and plethoric constitution, having sallied forth from a public house, half drunk, stumbled and fell with the right foot projecting outwards, and backwards, but sustaining the weight of the body in the fall. He made an effort to get up; but the pain and displacement of the foot rendered walking impossible, and he was carried to the Hotel Dieu, on the 28th of February, 1816."

Here M. Dupuytren soon recognized all the signs of fracture of the fibula, as deviation and rotation of the foot outward, projection of the malleolus internus, depression and crepitation above the outer ancle, &c. But what attracted attention most were, the great distance between the two malleoli, the projection of the tibia to near the middle of the sole of the foot, and the ascent of the astragalus, external malleolus, and, in fact, of the whole foot, for a distance of two inches along the external face of the tibia, ("l'ascension de l'astragale, de la malleole peroneale, et de la totalité du pied le long de la face externe du tibia jusqu' à deux pouces de hauteur,") unusual phenomena in fracture of the tibia.

"The tumefaction, tension and pain, were augmenting every moment, and the patient was urgent for a reduction of the dislocation; but the contraction and spasms of the muscles rendered this impossible, till venesection was employed, when by renewed efforts a reduction was effected."

The apparatus of Dupuytren kept the parts in situ, and a cure was effected, in the short and almost incredible space of thirty-six days.

*Treatment.* When the fibula is fractured at more than three inches from the malleolus externus, and without any displacement, it requires only *rest* to prevent pain, swelling, and other accidents; and *time* to allow the bones to unite. Those fractures which are situated *within* three inches of the extremity of the bone, require perfect rest and security from motion, in a much more imperious manner than the preceding accident; and this too, where there is no dislocation of parts, in order to prevent such a serious event. Fractures of the fibula, with simple dislocation of the foot, require *immediate* reduction; and still more imperiously so, when the fracture and dislocation are accompanied with rupture of the ligaments, separation of the malleolus externus, fracture of the tibia, extravasations of blood, wounds of the integuments, &c.

M. Dupuytren conceives that no question can arise, as to the propriety of *immediate* reduction, when a surgeon is called to the patient *immediately* after the accident occurs; but he observes, that the propriety of this measure has been doubted, when some hours have elapsed, and when tumefaction, tension, strangulation, and inflammation, have supervened. M. Dupuytren decides, and we think justly, that reduction is to be immediately put in force at whatever period we are called in to the accident. In order to illustrate the danger of waiting, in such cases, for a dissipation of the swelling and inflammation, our author states the following case, taken from among a great number of similar examples.

" A man being mounted in a pear tree, the branch on which he sate, broke, and he fell on the inner edge of the right foot. He immediately experienced an acute pain in the ancle joint, quickly followed by considerable tumefaction of the parts. A country surgeon being called, considered the affair as only a sprain, and confined his measures to some topical applications, and blood-lettings. But a violent fever being kindled up, with spasms and delirium, another surgeon was consulted on the fifth day from the accident, who, notwithstanding the tumefaction, easily detected a fracture of the fibula, and a displacement of the foot *outwards*. Still it was decided to wait a reduction of the inflammation, before attempting a reduction of the dislocation. Under this plan, several points of the surface were threatened with gangrene—an abundant suppuration manifested itself round the joint—and M.Dupuytren was called in in consultation.

" Struck with the prominency of the malleolus internus and astragalus inwards, the deviation of the foot outwards, the depth of the depression above the inner ancle, and the severity of the attendant symptoms, M. Dupuytren proposed an immediate reduction, in order to remove the primary cause of all these bad symptoms. But he was over-ruled, and the proposal deemed by his colleagues to be dangerous and unnecessary. Meanwhile, large sloughs began to form opposite to the projecting malleolus internus, and over the fractured fibula, and the cellular tissue under the skin became the seat of extensive suppurations. At the end of three weeks, the violence of the symptoms having subsided, the proper time for reduction appeared to have arrived. Extension and counter-extension were made with excessive pain, but very little effect; and a common apparatus for fracture of the leg was applied. Vain efforts! The foot could not be kept in its proper place; whether from the inefficiency of the apparatus, or the swelling and disorganization of the parts. During the five following days, these attempts at reduction and retention, in situ, were repeatedly made, with little or no success, and were ultimately given up. The patient having nearly perished more than once, from extensive erysipelas, bilious fever, excessive suppurations, and colliquative perspirations, at length recovered, but with the limb completely distorted, and, of course, with lameness for life." P. 174.

Our author here remarks that, in reality, the reduction of these fractures is seldom impracticable during the first week or two of the accident; and, that even in the above instance, it might have been effected by a proper apparatus.

*Reduction.* M.Dupuytren truly observes, that the ends of fractured bones can have no tendency to separate from one another—this tendency can only result from the action of neighbouring muscles. Here this able surgeon pays a just tribute to our illustrious Pott, whose principles, we are happy to see, are followed in the Hotel Dieu. Fractured and dislocated limbs are there laid in a state of demi-flexion, and not in the straight positions, which have been pretty generally chosen by the French surgeons, of late years. This relaxation of the muscles is, of course, a sine qua non in the reduction, as well as the retention.

We shall now describe M. Dupuytren's apparatus for dislocated ancle joints, and fractured fibula, which, as he observes, is of such simple mechanism, that it can easily be understood by the dullest apprehension—is composed of such common materials as are every where readily found—and is of such easy application, that it presents no difficulty, even to the most inexperienced tyro in surgery.*

A cushion, a splint, and two rollers compose the whole of the apparatus. The cushion, made of linen, and about two thirds filled of cotton, should be about two feet and a half [French] long, four or five inches broad, and three or four inches thick. The splint, from eighteen to twenty inches long, two and a half inches broad, and three or four lines in

* Mr. Alcock, surgeon, of Piccadilly, a gentleman of great talent, and of varied and pre-eminent acquirements, has favoured us with a plate of this apparatus, and permission to work off as many copies as we wanted for the present number. The drawing first appeared in the Medical Intelligencer for April last.

London, Published, March, 1820, by Mess.rs Burgess & Hill, No. 55, Great Windmill Street.

thickness, ought to be made of pretty firm wood, and but little elastic. One of those employed for fractures of the leg will do very well. The rollers, of half worn linen, should each be about four or five ells in length.

The cushion, being doubled upon itself, lengthwise, forms a kind of wedge, the thicker extremity of which is to rest on the malleolus internus, but not to pass that; while the other extremity comes on the internal condyle of the femur, thus furnishing a complete defence to the leg against the splint, and a *point d'appui* for the latter when the roller is applied. The splint then being laid along over the cushion, ought to pass the inferior extremity of the latter, from five to six inches, so as to project three or four inches beyond the inner edge of the foot itself. One of the rollers is now to be neatly wound round the leg and apparatus, below the knee, so as to secure it at that point; while the other roller being passed twice or thrice round the projecting portion of splint below, so as to take hold on it, is to be wound from thence round the heel and instep alternately, the vacant space between the splint and foot enabling the surgeon to draw the latter towards the former, by figure of 8 turns of the roller, with any degree of force that may be necessary to bring the foot into a proper line of bearing with the leg, and retain it there. By this apparatus, not only is the foot drawn inwards to its proper place by the roller going round the projecting splint as its lever, but the tibia and astragalus are pressed outwards by the base of the cushion, which forms the grand point d' appui of the whole apparatus. Thus, also, the lower fragment of the fibula being pressed *outwards* by the tibia above, and drawn *inwards* by the lateral ligaments below, exercises an action on the astragalus the reverse of that which displaced it, and consequently retains it in its natural situation. The limb is now to be half bent, and laid with its outside resting on a pillow, when the patient will generally express himself as being very easy and comfortable.*

The above apparatus is perfectly applicable to all cases of fracture of the fibula with dislocation of the foot, both outwards and inwards. If the foot be thrown *outwards*, the splint is to be placed on the inside of the leg—if *inwards*, on the outside. When the foot is thrown *forwards*, the apparatus will still answer, if placed along the fibula or tibia.

When the foot is thrown backwards, it is not only difficult to reduce it, on account of the strong action of the

* See the plate of this apparatus in the front of this number.

gastrocnemii and solei muscles, but it is also with difficulty retained, *in situ*, after reduction; for the superior surface of the astragalus, (being convex from behind forwards) is so slippery, that it is with much difficulty the tibia can be made to rest steady thereon. The tibia, in fact, has a constant tendency to slip forwards, while the astragalus is as constantly drawn backwards by the action of the extensor muscles.

M. Dupuytren, after trying a number of expedients, at length found, that the following plan was effectual, as the apparatus, at one and the same time, pressed the foot from behind forwards, and the tibia from before backwards;—the sine qua non, in all cases, being a half-bent position of the limb.

The apparatus before described, with the addition of a small cushion a few inches square, and stuffed with hair or cotton, is sufficient. In the dislocation in question, after reduction, the large cushion is to be placed *under* the limb, along the gastrocnemii muscles, reaching from the heel to the ham—its base or thicker end being below, and its slenderer extremity above. Over this cushion is to be laid the splint before described, and round the upper part of the leg and apparatus, a roller neatly applied. The small cushion being now placed on the fore part of the leg close to the instep, the second roller is to be carried round this and the lower part of the splint with such a degree of tightness as may be judged necessary. It is evident, that the action of this roller is to press the heel forward and the tibia backward at the same time; and this may be done with any degree of force that may be thought requisite to resist the tendency of the muscles to re-dislocate the parts.

In drawing a parallel between the apparatus for these accidents, now used by Dupuytren, and the older apparatus for the same, we are informed by our author, that an experience of two hundred and seven cases, treated on the new plan, has furnished abundant evidence of its vast superiority. It not only brings the parts that are fractured and dislocated into the most exact approximation, but it so retains them there, that after the cure is effected, not the slightest trace of deformity remains. It is equally applicable to the most comminuted fractures and the most simple dislocations. By retaining the parts exactly in their natural situation, it obviates a vast deal of inflammation, by instantly removing the pain and distraction of parts:—It, consequently, in this way, prevents a number of constitutional symptoms also, as spasms, fever, tetanus, &c. which are often fatal in their results.

We have thus endeavoured to give a very full Analysis, or rather, indeed, a concentrated translation of this important paper, well knowing that we are thereby conferring a greater benefit on our English readers, than if we attempted to pourtray half a dozen of articles in the same space, but without leaving a single complete picture on the mental optics. It is a little curious that the Dupuytren of London, and the Cooper of Paris should have published, at nearly the same moment, on Dislocations of the Ancle Joint. They have enabled us, in two successive numbers of this Journal, to lay before all ranks of the profession, the most valuable series of observations on this interesting point of Surgery that ever appeared in any age or country. We conceive that in such instances as these we best consult our own duty and the public good, by expending our labour in faithful analysis rather than in fastidious criticism.

——————— quæ non fecimus ipsi
Vix ea nostra voco.

## Figures and Tables

**Figure f1:**